# Supplementary material for: Hot topic: Mapping of the human intranasal mucosal thermal sensitivity: A clinical study on thermal threshold and trigeminal receptors
Source: PLoS One. 2024 Aug 6;19(8):e0304874. doi: 10.1371/journal.pone.0304874 (PMC11302907; doi:10.1371/journal.pone.0304874)
Supplement: S1 File — (DOCX) [file pone.0304874.s001.docx]

**Supplementary information**

mRNA expression of receptor types and their distribution normalized to PPIA

For the ratio of the receptor expression to PPIA the same results were found with no main effect for the sites (F(2)=0.30, p=0.74), but a main effect for the receptors (F(1.02)=126.95, p<0.001) and no interaction effect (F(1.89)=0.61, p=0.54).

For the relative expression of TRPV1 normalized to PPIA the same results emerged with no effect for the site (F(2)=0.047; p=0.95) nor a difference in the pairwise comparison (p=1.00 for all pairs).

For the relative expression of TRPV3 normalized to PPIA there was a difference regarding the site (F(1.03)=13.49; p<0.001; Greenhouse-Geisser). In the pairwise comparison, a higher TRPV3 expression for the anterior septum compared to the middle turbinate (p=0.004, *M*Diff = 0.002, 95%-CI[0.001-0.003]) and the olfactory cleft (p=0.005, *M*Diff = 0.002, 95%-CI[0.000-0.003]) was shown.

The relative expression of TRPA1 normalized to PPIA showed no effect for the site (p=0.943) and no difference in a pairwise comparison (p=1.00 for all pairs). Regarding the relative expression of TRPM8 to PPIA there was no effect for the site (p=0.68).

Correlation of all different tests

**Within the psychophysical tests,** a negative correlation was found for the TDI score and AmmoLa (r=-0.58, p=0.007). For the TDI score and CO_2_ threshold, a trend of a negative correlation was found (r=-0.403, p=0.078), showing an association between a higher TDI score and a lower CO_2_ threshold. For the lateralization and the TDI score, no such correlation was found (p=0.46). Further, there was a positive correlation between the lateralization test and AmmoLa ratings (r=0.511, p=0.021).

Regarding the **CO_2_ threshold**, a negative correlation was observed for the subjective rating of nasal patency in the right nostril (r=-0.444, p=0.050), but not in the left nostril or both nostrils.

**Within the rating of the nasal patency**, there was a positive correlation between the nasal patency of the left side and both sides (r=0.72, p<0.001). There were negative correlations between the nasal patency of the left site and the thermal threshold at the nose tip (r=-0.61, p=0.004) respectively lower turbinate (r=-0.45, p=0.049). For the nasal patency of both sites, there were negative correlations to the thermal threshold of the anterior septum (r=-0.48, p=0.034) and the nose tip (r=-0.60, p=0.005).

*Table 4: Data generated and analyzed during this study.*

| Participant | gender [1 female, 2 male] | age [years] | height [cm] | weight [kg] | TDI | Lateralisation [20 trials] | CO2 threshold [ms] | AmmoLa stick | Nasal patency left | Nasal patency right | Nasal patency both | Questionnaire trigeminal function | Stimulation site [1 right, 2 left] | Anterior septum_threshold [ms] | Lateral vestibulum threshold [ms] | Nose tip threshold [ms] | Lower turbinate threshold [ms] | Middle septum threshold [ms] | Middle turbintae threshold [ms] | Olfactory cleft threshold [ms] |
| --- | --- | --- | --- | --- | --- | --- | --- | --- | --- | --- | --- | --- | --- | --- | --- | --- | --- | --- | --- | --- |
| 1 | 1 | 22 | 162 | 57 | 31,25 | 20 | 772 | 7,5 | 5,4 | 9,8 | 8 | 25 | 1 | 1350 | 1400 | 600 | 650 | 550 | 450 | 350 |
| 2 | 1 | 21 | 165 | 60 | 36,75 | 20 | 790 | 7,5 | 8 | 9 | 9 | 28 | 1 | 950 | 650 | 800 | 225 | 13 | 70 | 138 |
| 3 | 1 | 21 | 175 | 68 | 36 | 20 | 1290 | 6,8 | 7 | 8 | 7,5 | 26 | 2 | 1575 | 145 | 550 | 450 | 750 | 425 | 95 |
| 4 | 1 | 22 | 168 | 70 | 32,5 | 20 | 1966 | 9,5 | 8,5 | 7,2 | 8,5 | 22 | 1 | 700 | 825 | 1075 | 275 | 275 | 250 | 350 |
| 5 | 2 | 22 | 195 | 75 | 34,5 | 17 | 1956 | 4 | 9,5 | 8,5 | 9 | 24 | 2 | 2600 | 225 | 175 | 200 | 300 | 200 | 250 |
| 6 | 2 | 23 | 191 | 89 | 33 | 12 | 846 | 7 | 8,3 | 7 | 7,8 | 21 | 2 | 1925 | 800 | 875 | 675 | 750 | 550 | 1350 |
| 7 | 2 | 22 | 185 | 87 | 34 | 9 | 1935 | 6,75 | 6 | 8,5 | 7 | 27 | 1 | 2275 | 450 | 1525 | 450 | 1025 | 1325 | 1450 |
| 8 | 1 | 24 | 172 | 65 | 37,25 | 19 | 1532 | 8 | 7,5 | 7,5 | 8 | 27 | 1 | 825 | 150 | 750 | 225 | 550 | 325 | 675 |
| 9 | 2 | 23 | 183 | 75 | 30,5 | 19 | 1439 | 9,5 | 7,2 | 8 | 7,8 | 13 | 1 | 3900 | 1350 | 1475 | 3075 | 1800 | 2825 | 1225 |
| 10 | 2 | 23 | 189 | 78 | 34,25 | 19 | 1873 | 6,4 | 7,8 | 7,8 | 7,8 | 24 | 2 | 350 | 450 | 350 | 750 | 550 | 550 | 325 |
| 11 | 1 | 24 | 161 | 54 | 37,5 | 16 | 199 | 6 | 9,2 | 8,9 | 9 | 21 | 2 | 425 | 550 | 400 | 625 | 350 | 250 | 150 |
| 12 | 1 | 26 | 165 | 56 | 31,75 | 20 | 1594 | 10 | 7 | 7 | 10 | 27 | 2 | 225 | 1325 | 925 | 1050 | 1050 | 600 | 250 |
| 13 | 2 | 23 | 182 | 86 | 31 | 20 | 1935 | 10 | 9 | 9 | 9 | 17 | 1 | 2200 | 450 | 600 | 750 | 1800 | 1875 | 1350 |
| 14 | 1 | 23 | 193 | 75 | 34 | 20 | 1935 | 9,7 | 9,5 | 9,5 | 9,5 | 24 | 2 | 575 | 550 | 550 | 825 | 525 | 300 | 450 |
| 15 | 1 | 25 | 163 | 57 | 33,75 | 19 | 509 | 8 | 9 | 9 | 10 | 24 | 2 | 200 | 200 | 100 | 150 | 200 | 200 | 200 |
| 16 | 2 | 21 | 186 | 66 | 33,75 | 20 | 695 | 9,5 | 3,8 | 9,5 | 7,5 | 27 | 1 | 100 | 350 | 1150 | 1800 | 800 | 350 | 200 |
| 17 | 2 | 22 | 178 | 75 | 36,75 | 18 | 540 | 6,8 | 9,8 | 9,8 | 9,8 | 19 | 1 | 150 | 150 | 100 | 150 | 150 | 150 | 100 |
| 18 | 1 | 22 | 180 | 70 | 31,5 | 20 | 1935 | 9,8 | 8,5 | 7,3 | 9,5 | 27 | 1 | 550 | 600 | 350 | 350 | 950 | 375 | 1025 |
| 19 | 1 | 27 | 167 | 58 | 35,5 | 16 | 1067 | 6 | 10 | 10 | 10 | 21 | 2 | 150 | 150 | 125 | 375 | 150 | 150 | 150 |
| 20 | 2 | 26 | 186 | 95 | 35 | 16 | 1439 | 5 | 10 | 7,5 | 10 | 27 | 2 | 750 | 350 | 750 | 400 | 150 | 300 | 1500 |

*Continuation of Table 4: Data generated and analyzed during this study. The receptor expression in relation to TBP is specified with 4 decimal places.*

| Participant | Anterior septum TRPV1/TBP | Anterior septum TRPV3/TBP | Anterior septum TRPA1/TBP | Anterior septum TRPM8/TBP | Middle turbinate TRPV1/TBP | Middle turbinate TRPV3/TBP | Middle turbinate TRPA1/TBP | Middle turbinate TRPM8/TBP | Olfactory cleft  TRPV1/TBP | Olfactory cleft  TRPV3/TBP | Olfactory cleft  TRPA1/TBP | Olfactory cleft  TRPM8/TBP |
| --- | --- | --- | --- | --- | --- | --- | --- | --- | --- | --- | --- | --- |
| 1 | 0,1972 | 0,2246 | 0,0000 | 0,0000 | 0,3911 | 0,0000 | 0,0000 | 0,0000 | 0,3586 | 0,0063 | 0,0045 | 0,0021 |
| 2 | 0,3165 | 0,0914 | 0,0000 | 0,0000 | 0,3882 | 0,0048 | 0,0000 | 0,0000 | 0,3544 | 0,0030 | 0,0000 | 0,0000 |
| 3 | 0,2278 | 0,0091 | 0,0003 | 0,0008 | 0,4061 | 0,0036 | 0,0010 | 0,0000 | 0,2406 | 0,0012 | 0,0000 | 0,0010 |
| 4 | 0,2178 | 0,0291 | 0,0000 | 0,0000 | 0,2857 | 0,0067 | 0,0007 | 0,0021 | 0,3587 | 0,0009 | 0,0000 | 0,0000 |
| 5 | 0,2732 | 0,0148 | 0,0000 | 0,0000 | 0,2594 | 0,0040 | 0,0000 | 0,0000 | 0,2312 | 0,0005 | 0,0022 | 0,0000 |
| 6 | 0,3349 | 0,0420 | 0,0000 | 0,0000 | 0,1341 | 0,0072 | 0,0004 | 0,0000 | 0,2451 | 0,0026 | 0,0006 | 0,0000 |
| 7 | 0,3413 | 0,0445 | 0,0000 | 0,0000 | 0,1553 | 0,0040 | 0,0002 | 0,0000 | 0,2365 | 0,0246 | 0,0000 | 0,0000 |
| 8 | 0,3887 | 0,0030 | 0,0000 | 0,0000 | 0,1966 | 0,0011 | 0,0004 | 0,0011 | 0,2763 | 0,0017 | 0,0000 | 0,0012 |
| 9 | 0,3624 | 0,0278 | 0,0000 | 0,0000 | 0,3738 | 0,0022 | 0,0000 | 0,0000 | 0,2141 | 0,0223 | 0,0000 | 0,0000 |
| 10 | 0,1791 | 0,0151 | 0,0000 | 0,0000 | 0,3371 | 0,0007 | 0,0000 | 0,0026 | 0,3674 | 0,0022 | 0,0000 | 0,0000 |
| 11 | 0,6435 | 0,0263 | 0,0000 | 0,0000 | 0,3238 | 0,0009 | 0,0010 | 0,0011 | 0,2498 | 0,0032 | 0,0003 | 0,0007 |
| 12 | 0,1913 | 0,0287 | 0,0000 | 0,0029 | 0,2524 | 0,0060 | 0,0009 | 0,0000 | 0,4466 | 0,0059 | 0,0013 | 0,0000 |
| 13 | 0,1280 | 0,0432 | 0,0000 | 0,0000 | 0,2821 | 0,0041 | 0,0002 | 0,0000 | 0,1319 | 0,0129 | 0,0000 | 0,0000 |
| 14 | 0,2348 | 0,0199 | 0,0000 | 0,0023 | 0,1757 | 0,0058 | 0,0000 | 0,0011 | 0,1440 | 0,0043 | 0,0000 | 0,0003 |
| 15 | 0,2372 | 0,0462 | 0,0000 | 0,0000 | 0,1328 | 0,0078 | 0,0005 | 0,0006 | 0,1823 | 0,0102 | 0,0013 | 0,0023 |
| 16 | 0,2390 | 0,0308 | 0,0000 | 0,0012 | 0,3929 | 0,0004 | 0,0006 | 0,0000 | 0,2217 | 0,0035 | 0,0005 | 0,0012 |
| 17 | 0,1567 | 0,0340 | 0,0122 | 0,0000 | 0,2731 | 0,0073 | 0,0006 | 0,0000 | 0,2742 | 0,0062 | 0,0007 | 0,0009 |
| 18 | 0,2679 | 0,0204 | 0,0002 | 0,0006 | 0,3956 | 0,0005 | 0,0024 | 0,0000 | 0,2863 | 0,0023 | 0,0000 | 0,0000 |
| 19 | 0,3473 | 0,0116 | 0,0000 | 0,0000 | 0,2430 | 0,0053 | 0,0000 | 0,0000 | 0,4225 | 0,0031 | 0,0000 | 0,0000 |
| 20 | 0,1757 | 0,0265 | 0,0000 | 0,0042 | 0,2201 | 0,0034 | 0,0000 | 0,0000 | 0,1986 | 0,0067 | 0,0000 | 0,0000 |
