# Supplementary material for: Hot topic: Mapping of the human intranasal mucosal thermal sensitivity: A clinical study on thermal threshold and trigeminal receptors
Source: PLoS One. 2024 Aug 6;19(8):e0304874. doi: 10.1371/journal.pone.0304874 (PMC11302907; doi:10.1371/journal.pone.0304874)
Supplement: Attachment — Submitted filename: Letter_23-12-6.docx [file pone.0304874.s002.docx]

**Susanne Weise**, MD
Smell & Taste Clinic, Department of Otorhinolaryngology

Fescherstraße 74

01307 Dresden

Germany

December 6^th^, 2023

Dear Editors,

I appreciate the time and effort you and the reviewers have dedicated to evaluating my manuscript.

1. **financial disclosure:** The reviwers suggested detailed information regarding the role the funders took in the study. I confirm: “The funders had no role in study design, data collection and analysis, decision to publish, or preparation of the manuscript.”
2. **funding information in manuscript:** The reviewers suggested to remove any funding-related text from the manuscript. After thoroughly revising the text, I removed all funding-related information from the manuscript.
3. **response to Reviewers letter:** The reviewers recommended uploading the response to the reviewers' letter (in a file 'Response to Reviewers'), which should include a detailed response to each point raised by the Editor and/or Reviewers. This response has been submitted as a file titled 'Response to Reviewers.'
4. **data availability:** The reviewers requested the submission of the minimal dataset. A shortened dataset is included in the Supporting Information."
5. **supplementary tables are included in the manuscript: The reviewers recommended to remove supplementary tables from the manuscript.** After thoroughly revising the manuscript, I removed **supplementary tables.**
6. **link to Table 4 and 5:** The reviewers suggested to refer to Table 4 and 5 in the manuscript. After thoroughly revising the text, I removed the tables because a the data set will be provided.
7. **ethics statement:** The Reviewers emphasised that the ethics statement should only be written in the Methods section. Following the revision of the manuscript, the Following the revision of the manuscript, the appears only in the Methods section.

I would like to express my gratitude for the constructive feedback provided by the reviewers. Their insights have been invaluable in enhancing the quality of the manuscript.

Thank you for your consideration. I look forward to hearing your thoughts on the revised manuscript.

Best regards,

Susanne Weise, Susanne.weise@uniklinikum-dresden.de (corresponding author)
