# Supplementary material for: Hot topic: Mapping of the human intranasal mucosal thermal sensitivity: A clinical study on thermal threshold and trigeminal receptors
Source: PLoS One. 2024 Aug 6;19(8):e0304874. doi: 10.1371/journal.pone.0304874 (PMC11302907; doi:10.1371/journal.pone.0304874)
Supplement: Attachment — Submitted filename: point-by-point reply_reviewer_2024-03-12.doc [file pone.0304874.s003.doc]

**PONE-D-23-24390R1**

**“Hot topic: Mapping of the human intranasal mucosal thermal sensitivity: A clinical study on thermal threshold and trigeminal receptors” by Menzel et al.**

**Author´s reply to the comments of the reviewer**

The authors thank the referees for the constructive criticism to which we would like to respond as follows:

1. *It is not clear to me what could be the physiological meaning of testing thermal sensitivity in the nose. The authors might want to better explain the rationale of the study.*

Answer:

The authors thank the reviewer for the comments. The olfactory and trigeminal system are closely interlinked in terms of anatomy and function. Trigeminal activation can be induced by thermal, mechanical, or chemosensory stimulation. While numerous studies have concentrated on mechanical and chemosensory trigeminal stimulation, there exists a gap in information concerning thermal trigeminal stimulation. This study seeks to fill this void by shedding light on the functional and physiological aspects of thermal stimulation within the nasal cavity.

In this study, the thermal stimulation in the human nasal cavity was conducted for the first time. The stimulus threshold was investigated at various points within the nose. Additionally, the perception of suprathreshold stimulation was evaluated. Furthermore, nasal swabs were taken to examine receptor expression for the perception of thermal stimuli at different points within the nasal cavity.

The knowledge gained is important from a scientific perspective due to the inaugural exploration of thermal stimulation in the nasal cavity. Furthermore, these findings might be applied in the future for therapeutic trigeminal stimulation within the nose, for trigeminal dysfunction or olfactory dysfunction due to the close interlink between both systems.

2. *Since the most relevant and innovative aspect of the paper is indeed the nasal thermal stimulation, the authors should better explain the stimulation protocol. Indeed, it is not clear to me how the stimulation worked. For example: Could the authors apply different temperatures (from figure 2 it seems that this is the case but the discussion section lines 438 to 440 “thermal stimulation at various temperatures and for extended stimulating intervals could provide a more comprehensive characterization of perception” confused me)? In addition, how are duration and intensity related? I could not understand it from the methods section and figure 2 do not help in clarifying this issue.*

Answer:

The authors thank the reviewer for these comments. In response to the comment, the Methods section has been revised to clearly elucidate the procedure for thermal stimulation, see line 163-207.

3. *A somehow related topic is the consideration of the thermal stimulation as mechanical one. This is reported in the discussion (line 426-431). Unless better explained I would consider thermal stimulation as different from mechanical. TRP channels are rather poorly sensitive to mechanical stimulation unless it is referred to a change in cellular volume. Do the authors mean that during heating the electrode went through thermal expansion? Would then be the expansion the mechanical stimulus? It is rather confusing; I think the issue could be solved by a clearer explanation of the set-up and protocol in the methods.*

Answer:

The authors thank the reviewer for these comments. The reviewers are absolutely correct in pointing out the distinction between thermal and mechanical stimulation in this study. In the present study, the thermal stimulation was administered via an electrode that needs to be placed on the tissue. By positioning the electrode on the intranasal mucosa, discrete irritation can occur, which might result in a discrete mechanical stimulus. However, the placement of the electrode was done under endoscopic control in order to decrease the potential mechanical stimulation. We did not consider a thermal expansion due to the short exposure to heat. The potential mechanical irritation mentioned in the discussion (line 430) refers solely to the process of electrode placement on the tissue. The section methodology was revised to elucidate this aspect (see line 186-188).

This study is the first one on thermal intranasal stimulation, emphasizing the significance of the study.

4. *I think the qPCR lack of positive controls, since the authors state that often they failed to find TRPA1 and TRPM8. How could they be sure that the qPCR run under optimal conditions and with the right primers?*

Answer:

The authors thank the reviewer for these comments. This study exclusively utilized assays validated by the manufacturer, which were cultivated under the conditions specified by the manufacturer. A positive control was conducted with the cell line LNCaP.

5. *The Discussion section is too long, shortening may help to make it clearer.*

Answer:

The authors thank the reviewer for these comments. The authors followed the reviewer’s suggestion, the section discussion was shortened and thoroughly revised (line 389-536).
